# The Role and Dynamics of β-Catenin in Precondition Induced Neuroprotection after Traumatic Brain Injury

**DOI:** 10.1371/journal.pone.0076129

**Published:** 2013-10-04

**Authors:** Gali Umschweif, Alexander G. Alexandrovich, Victoria Trembovler, Michal Horowitz, Esther Shohami

**Affiliations:** 1 Department of Pharmacology, The Hebrew University, Jerusalem, Israel; 2 Laboratory of Environmental Physiology, The Hebrew University, Jerusalem, Israel; Universidade de São Paulo, Brazil

## Abstract

Preconditioning via heat acclimation (34°C 30 d) results in neuroprotection from traumatic brain injury due to constitutive as well as dynamic changes triggered by the trauma. Among these changes is Akt phosphorylation, which decreases apoptosis and induces HIF1α. In the present study we investigated the Akt downstream GSK3β/β -catenin pathway and focused on post injury alternations of β catenin and its impact on the cellular response in preconditioned heat acclimated mice. We found that the reduction in motor disability is accompanied with attenuation of depressive like behavior in heat acclimated mice that correlates with the GSK3β phosphorylation state. Concomitantly, a robust β catenin phosphorylation is not followed by its degradation, or by reduced nuclear accumulation. Enhanced tyrosine phosphorylation of β catenin in the injured area weakens the β catenin-N cadherin complex. Membrane β catenin is transiently reduced in heat acclimated mice and its recovery 7 days post TBI is accompanied by induction of the synaptic marker synaptophysin. We suggest a set of cellular events following traumatic brain injury in heat acclimated mice that causes β catenin to participate in cell-cell adhesion alternations rather than in Wnt signaling. These events may contribute to synaptogenesis and the improved motor and cognitive abilities seen heat acclimated mice after traumatic brain injury.

## Introduction

Heat acclimation (HA, 34°C for 30 days) is a pre-conditioning model that confers protection against various kinds of stressors. After the acclimation period, HA animals cope better with insults due to reprogrammed gene expression and altered post transcriptional mechanisms [1]. Over the last decades we have shown that HA confers cardio protection against heat stress[2], ischemic insults [3] and hyperbaric oxygen [4]. Moreover, HA provides neuroprotection against central nervous system oxygen toxicity [5] and against traumatic brain injury (TBI) [6]. Investigation of the phenotypic changes involved in the neuroprotection in HA mice subjected to TBI revealed that activation of Akt by thr^308^ phosphorylation is an essential event [7]. Akt is a key protein in cellular pro-survival signaling including the activation of hypoxia inducible factor 1 (HIF-1) and the inhibition of the intrinsic apoptosis pathway. Indeed we have found that in HA mice, Akt activation is accompanied by decreased post injury apoptosis and induced HIF-1 activation that was recently shown to be indispensable for HA mediated neuroprotection [8]–[10]. Akt is a “check point” in numerous cellular pathways and has many down-stream effects, e.g. inhibition of glycogen synthase kinase 3β (GSK3β) by ser-9 phosphotylation. GSK3β was initially described as a major regulator of glycogen metabolism, however, following years of research it became clear that brain GSK3β activity also regulates other cellular processes by altering protein synthesis, cell proliferation, cell differentiation, cell motility and apoptosis [11]. GSK3β signaling has been linked to neuronal death in models of prion disease, stroke, Amyloid β neurotoxicity and of TBI [12]–[15]. Lately, it was documented that GSK3β mediates inflammation via the release of the pro-inflammatory cytokines IL1 and TNFα [16]. Importantly, the Akt/GSK3β pathway plays a role in post ischemic neuronal survival in other preconditioning models such as oxygen glucose depravation [17], [18]. However, GSK3β is not only important in cellular pathology after TBI, but also in the mental disorders related to TBI. It is a mediator of mood disorders including depression, a long lasting symptom of TBI [19]. GSK3β is also a key factor in canonical Wnt signaling activated by an extracellular Wnt ligand, and involved in the pathogenesis of various conditions. Wnt binds to the Frizzled receptor and to its co-receptors, the low-density lipoprotein receptor related proteins 5 and 6 (LRP5/6). Wnt binding inactivates a complex consisting of Adenomatosis polyposis coli (APC), Axin, casein kinase-1 (CK-1) and GSK-3β. Inactivated GSK3β allows β catenin to escape phosphorylation dependent proteosomal degradation, thereby promoting β-catenin-mediated gene transcription [20]. β catenin stability, determined by its phosphorylation state, is critical for cell survival and gliogenesis [21]. Other ser/thr kinases also regulate β catenin such as c-Jun N terminal kinase (JNK)[22]. Another major cellular role of β catenin is in cell-cell adhesion via its interaction with N-cadherin. The cell-cell adhesion is regulated by tyrosine phosphorylation of β catenin that leads to detachment of the cadherin catenin complex [21], [23]. Since β catenin stability and phosphorylation state determine the fate of the cell, they are finely regulated not only by phosphorylation but also by direct cleavage by proteases such as caspase 3 and calpain [24], [25]. It was also documented that hypothermia attenuates β catenin degradation after ischemia [26].

Since the HA model induces Akt phosphorylation, reduced apoptosis and transient hypothermia-all are involved in the determination of the fate of β catenin, this study was designed to investigate whether HA affects β catenin along one or both of its main pathways: (i) Wnt signaling (ii) cell-cell adhesion.

## Materials and Methods

### Animals and Maintenance

The study was approved by the Institutional Animal Ethics Committee of the Hebrew University and complied with the guidelines of the National Research Council Guide for the Care and Use of Laboratory Animals (NIH Publication no. 85-23, revised 1996). Male Sabra mice weighing 38 to 53g were used. Animals were kept under controlled light conditions with a 12 h/12 h light/dark cycle. Food and water were provided *ad libitum*. The mices were divided into two groups: control normothermic (NT) maintained at an ambient temperature of 24°C±1°C and heat acclimated (HA) held in a climatic chamber at 34°C±1°C for 30 days, a period that ensures acclimatory homeostasis has been achieved [1].

### Trauma Model

Experimental closed head injury (CHI), is a subtype of TBI without skull penetration. CHI was induced under isoflurane anesthesia using a modified weight drop device developed in our laboratory [27] Briefly, after anesthesia, a midline longitudinal incision was performed, exposing the skull. A Teflon tipped cone (2 mm diameter) was placed 1 to 2 mm lateral to the midline in the mid-coronal plane. The head was held in place and a 95 g weight was dropped on the cone from a pre-established height, resulting in focal injury to the left hemisphere. After recovery from anesthesia, the mice were given post-operative care in their cages with free access to food and water. Sham control mice underwent anesthesia and skin incisions.

### Neurobehavioral Evaluation

The functional status of the mice was evaluated according to the Neurological Severity Score (NSS) by an observer unaware of the treatment. This score is a 10 point scale assessing functional neurological status based on the presence of some reflexes and the ability to perform motor and behavioral tasks such as beam walking, beam balance and spontaneous locomotion [28]. Animals are awarded one point for failure to perform a task, i.e. scores increase with the severity of dysfunction. The NSS obtained 1 h post-CHI reflects initial injury severity, and the extent of the recovery (ΔNSS) can be calculated using the difference between the NSS at 1 h and at any subsequent time point. A positive ΔNSS indicates recovery, zero reflects no change and a negative ΔNSS indicates deterioration. NSS values were measured at 1 h, 24 h, 72 h and 7 days post-injury and ΔNSS was calculated at these times (n = 9–12 mice/group).

### Forced Swimming Test (FST)

In a calm quite room with an ambient room temperature of 25°C, mice were gently placed for 6 minutes in a cylindrical glass container (diameter 17 cm) filled with 24°C water at a depth of 22 cm. Mice were gently dried using towel and the water was changed after every test. Tests were recorded using a video camera and analyzed offline. Behavioral analysis was done from 2 to 6 minutes after placing the mouse in the water. Immobility was defined as minimal tail and limb movements to keep head above water. Mobility was defined as any movement other than those necessary to keep head above water. In order to avoid false results due to physical disability, only mice with moderate injury (NSS≤7) were subjected to the test (n = 7–9 mice/group).

### Western Immunoblotting

After 30 days of heat-acclimation or normothermic conditions, mice were subjected to CHI or sham surgery and sacrificed 6, 12, 24, 72 h or 7 days later (n = 5–6/group). After decapitation, brains were rapidly removed and frontal segments (40–60 mg) from left, injured hemispheres were separated and frozen at −80°C until analysis. Sample preparation was performed as previously described [8] with minor modifications. Nuclear and cytosolic extracts were prepared using a commercial kit (NE-PER, Pierce by Thermo Scientific labs Rockford, IL, USA). Validation of nuclear enrichment was conducted using α tubulin antibody (1∶2000; Sigma Aldrich St. Louis, MO, USA), that was almost undetected in the nuclear fraction. Some of the samples underwent further fractionation to obtain enriched membrane proteins fraction as previously described [29]: after homogenization in a buffer containing sucrose 0.25 M, Tris 20 mM (pH = 7.6), MgCl_2_ 1.5 mM, glycerol 10%, EDTA 1 mM, samples were centrifuged at 5000 rcf for 10 min. the supernatants were then homogenized for 30 min at 30,000 rcf and pellets were re-suspended in 40 µL homogenization, and used for membrane protein enriched subcellular fraction. Validation of membrane enrichment was conducted using anti synaptophysin (1∶2000; Merck Millipore, Billerica, MA, USA) that was almost absent in cytosolic extraction. After cellular fractionation, homogenates were stored at −80°C until analysis. Protein concentration was determined using the Bradford method (BioRad Labratories, Munich, Germany). Equal protein samples (40 µg for cytosolic and 20 µg for nuclear extracts) were separated on 10% SDS-polyacrylamide gels with 4.5% SDS stacking gels and electrotransferred onto nitrocellulose membranes (0.2 µm, Schleicher and Schuell, Dessel, Germany). Blots were probed with a rabbit polyclonal anti–GSK3β (1∶1000; cell signaling technology Inc., Danvers, MA, USA), anti-p-GSK3β^ser9^ (1∶1000; cell signaling technology Inc., Danvers, MA, USA), anti-β catenin (1∶1000; cell signaling technology Inc. Danvers, MA, USA), anti-p-β catenin^ser33/37/Thr41^,, anti JNK (1∶1000; cell signaling technology Inc., Danvers, MA, USA) or anti p-JNK ^Thr183/Tyr185^ antibody (1∶1000; cell signaling technology Inc., Danvers, MA, USA). Rabbit polyclonal antibody against β -actin antibody (1∶1000; cell signaling technology Inc. Danvers, MA, USA) was also used to confirm equal protein loading (Xilouri and Papazafiri 2006). Appropriate peroxidase-coupled immunoglobulin G (1∶5,000; Jackson Immunoresearch, Soham, Cambridgeshire, UK) was used for secondary incubations. Reactive bands were visualized using the enhanced chemiluminescence system (Biological Industries, Beit Haemek, Israel). Optical density of reactive bands was quantified using Tina software (Raytest, Straubenhardt, Germany) and protein levels were expressed as the optical density of the examined factor relative to β -actin in the same lane.

### Co-immunoprecipitation

The co-immunoprecipitation (co-ip) method was used to determine β catenin-N cadherin interactions and y654 phosphorylation of β catenin. Mice were subjected to CHI, and sacrificed 6, 12 or 24 h later. Brains were rapidly removed and frontal segments from the injured left hemisphere were homogenated in buffer containing 0.025 M Tris, 0.15 M NaCl, 0.001 M EDTA, 1% NP-40, 5% glycerol, pH = 7.4. Protein concentrations were determined using the Bradford method (BioRad Labratories, Munich, Germany) and homogenates were kept at −80°C until analysis. For co-ip, 200 µg protein in 200 µl was incubated overnight at 4°C with rotation with anti β catenin antibody (1∶200 cell signaling technology Inc. Danvers, MA, USA). 30 µl of cleaned Protein Immobilized rProtein (IPA-400HC, Repligen Waltham, MA, USA) was added and incubated with rotation for 30 minutes at 4°C. Following 3 minutes centrifugation at 14,000 rpm the pellet was washed five times with homogenization buffer. Finally sample buffer was added, samples were centrifuged at 12,000 rpm for 5 minutes and supernatants were collected. Western blot analysis was performed using either β catenin^tyr654^ antibody (1∶250, abcam Cambridge, UK),N-cadherin (1∶500; Abcam, Cambridge, UK), or anti β catenin antibody (1∶1000 cell signaling technology Inc. Danvers, MA, USA).

### Immunohistohemistry

At 24 h post injury, brains were perfused and fixed and 10 µm frozen sections were prepared. Slices were cut in the injured area (bregma +0.26). Brain slices were double stained for immunohistochemical evaluation using specific antibodies for β catenin tyr654 (1∶100, abcam Cambridge, UK) and DAPI (Sigma Aldrich St. Louis, MO, USA). Alexa 488 was used as the secondary antibody (1∶200, Molecular Probes, Leiden, The Netherlands). Pictures were taken using an epifluorescent Olympus microscope.

### Statistical Analysis

For statistical analyses, we used commercially available computer software (SigmaStat 2.03). Treatments were the independent variables and the outcomes of the TBI parameters were the dependent variables. Significance was tested using two-way ANOVA for repeated measures in NSS, followed by Tukey post-hoc tests. For protein levels and forced swimming test, significance was calculated using two-way ANOVA between HA and NT, or one-way ANOVA within the same group (NT or HA) followed by Tukey post-hoc tests. *P*-values<0.05 were considered significant for all comparisons. Data are expressed as mean ± s.e.m.

## Results

### Heat Acclimation Mediates Neuroprotection and Reduces Post Traumatic Depressive-like Behavior

**Figure 1:**
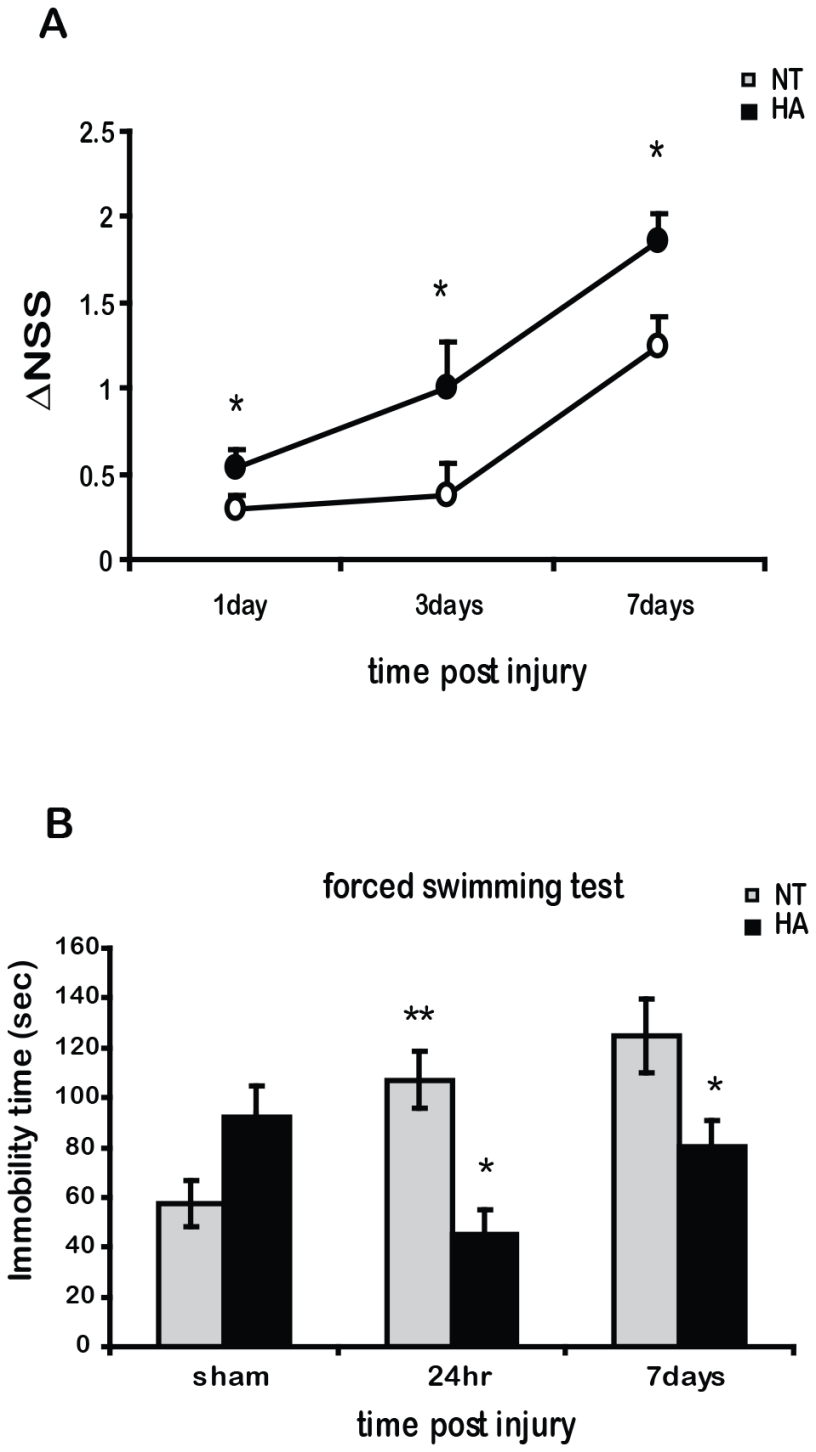
Heat acclimation (HA) induces improved motor recovery after TBI. Mice were subjected to CHI and motor ability was evaluated by the neurological severity score (*NSS*) at 1 h post injury for initial disability assessment and 1, 3 and 7 days thereafter. *ΔNSS* represent the recovery of injured mice as measured between 1 h post injury and any later time point, n = 9–12 per group (***A***). **Heat Acclimation (HA) reduces depressive like behavior after CHI**. Mice were subjected to CHI or sham operation and 24 h or 7 days thereafter to the forced swimming test. Immobility time was determined out of 240 seconds of record (***B***). Values represent the mean ± SEM. n = 5–6 per group. *p<0.05 vs. normothermic (NT) mice, **p<0.05 vs. sham mice within the same group.

HA mice recovered significantly better than NT controls after TBI (Figure 1A), signified by a greater ΔNSS 1, 3 and 7 days after injury. While TBI induced depressive like behavior in NT mice with increased immobility time in the forced swimming test (Figure 1B) 24 h and 7 days after injury, no such an effect was observed in the HA mice. HA mice exhibited reduced depressive like behavior and significantly shorter immobility times at 24 h after the injury and again at 7 days after the injury as compared to NT mice.

### HA Induces Inhibition of GSK3β and JNK after TBI

**Figure 2:**
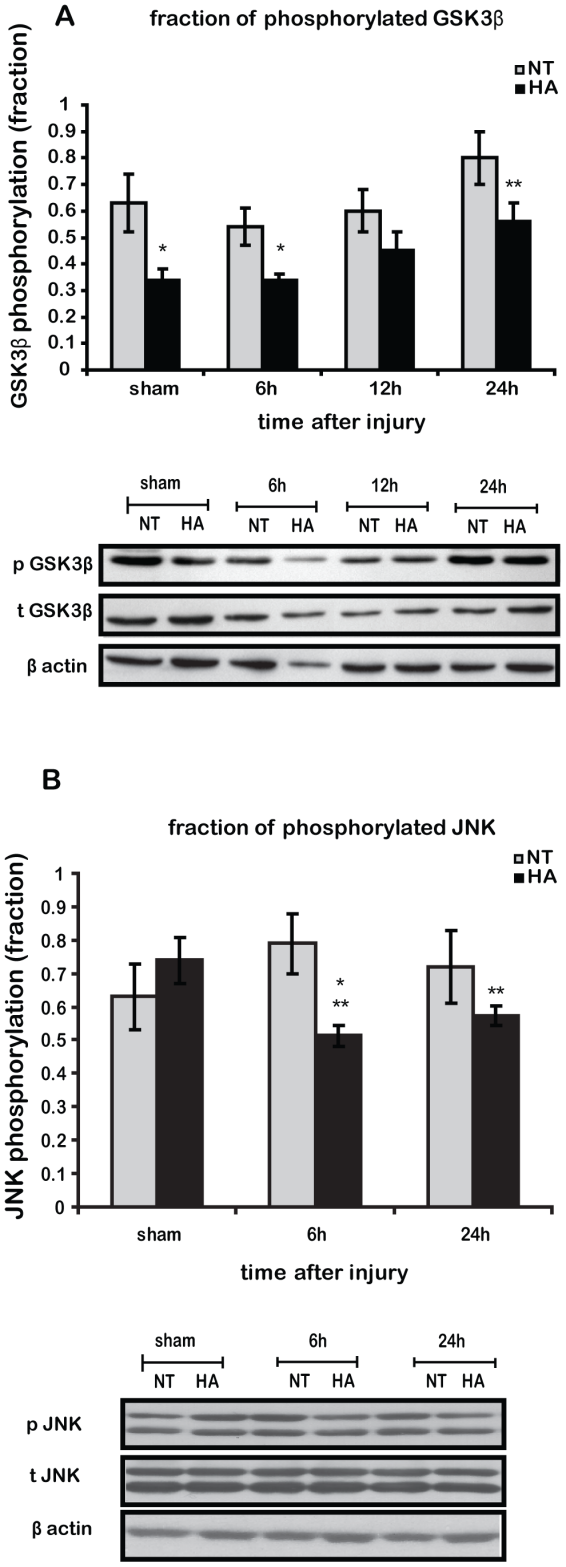
HA induces inhibition of glycogen synthase kinase 3β (GSK3β) and c-Jun N terminal kinase (JNK). Mice were subjected to CHI or sham operation, and injured cortexes were removed at 6, 12 or 24β showing significant inhibition of GSK3β by 24 h post injury in heat acclimated mice (HA) as compared with normothermic mice (NT) (***A***). Representative western blots of JNK phosphorylation, that is related to enhanced kinase activity, showing reduced JNK activity in HA mice after CHI (***B***). Values represent the mean ± SEM. n = 5–6 per group. *p<0.05 vs. NT mice, **p<0.05 vs. sham HA mice.

Since we observed HA induced behavioral changes and Akt phosphorylation, our next step was to evaluate the Akt downstream target and depression mediator GSK3β using Western blot analysis. We found that non injured sham HA mice have lower levels of non-activated p-GSK3β (higher ratio p-GSK3β/t-GSK3β) than the NT control mice.There was a significant increase in p-GSK3β 24 h after injury in HA mice, hence HA induces GSK3β inactivation after TBI, (Figure 2A). No significant changes were found in the total GSK3β levels in any group or treatment (data not shown). Another kinase involved in apoptosis and in β catenin phosphorylation is JNK. The basal phosphorylated, thus active, JNK fraction (p-JNK/t-JNK), was unchanged by HA alone. The injury did not affect the p-JNK fraction in NT mice, however JNK was significantly inactivated shortly after injury in HA mice and remained attenuated 24 h post injury (Figure 2B). No significant changes were found in the total JNK levels in any group or treatment (data not shown).

### Heat Acclimation Induces β Catenin Phosphorylation after TBI with no Consequent Alternation in Total or Nuclear Levels of β Catenin

**Figure 3:**
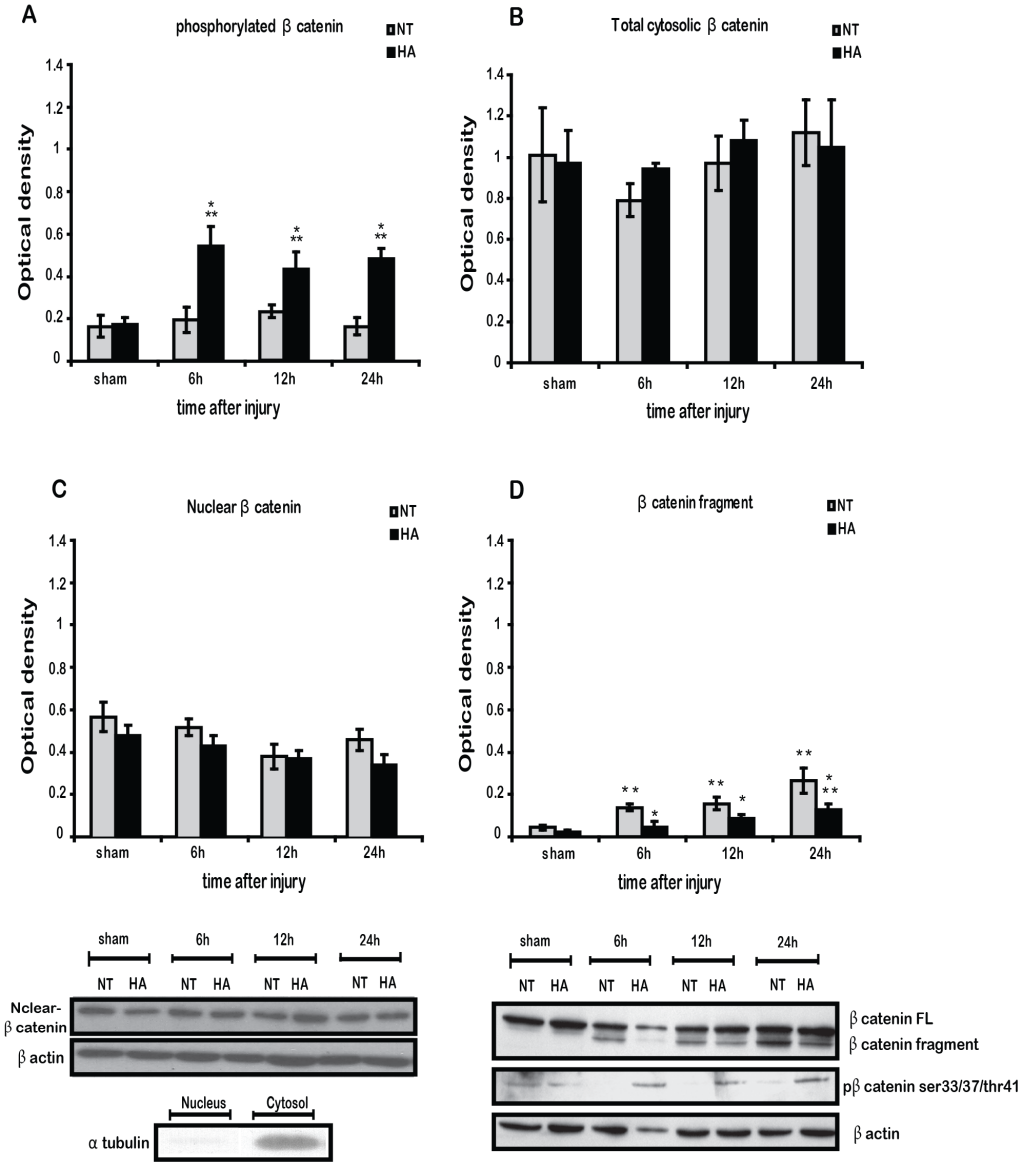
Heat acclimation (HA) enhances ser33/37thr/41 phosphorylation of β catenin without change in its nuclear/cytosolic levels. Mice were subjected to CHI or sham operation, and injured cortexes were removed at 6, 12 or 24/37thr/41 phosphorylation of β catenin is induced in injured HA mice (***A*** *)*. Total cytosolic fool length (*FL*) β catenin remain unchanged (***B***). Total nuclear β catenin remain unchanged. Anti α tubulin (1∶2000) was used to verify the absence of cytosolic proteins (***C***). 70–80 kD cytosolic β catenin fragment is significantly elevated after CHI in normothermic (NT) mice (***D***). Values represent the mean ± SEM. n = 5–6 per group. *p<0.05 vs. NT mice, **p<0.05 vs. sham HA mice within the same group.

After examination of the phosphorylation state of GSK3β and JNK, we examined their phosphorylation target, β catenin. TBI led to robust phosphorylation of β catenin at residues ser33/37thr44 6 h after injury. β catenin phosphorylation lasted up to 24 h post injury (Figure 3A). TBI did not affect p- β catenin in NT mice and those levels remained significantly attenuated compared to HA mice at all time-points after injury. Interestingly, no further degradation of β catenin was observed in HA mice following the intense phosphorylation, as its cytosolic levels remained unchanged throughout the experiment (Figure 3B). Also, nuclear levels of β catenin tended to decrease insignificantly in NT and HA mice after injury (Figure 3C).

### Heat Acclimation Induces Decreased Cleavage and Increased Tyrosine Phosphorylation of β Catenin after TBI

**Figure 4:**
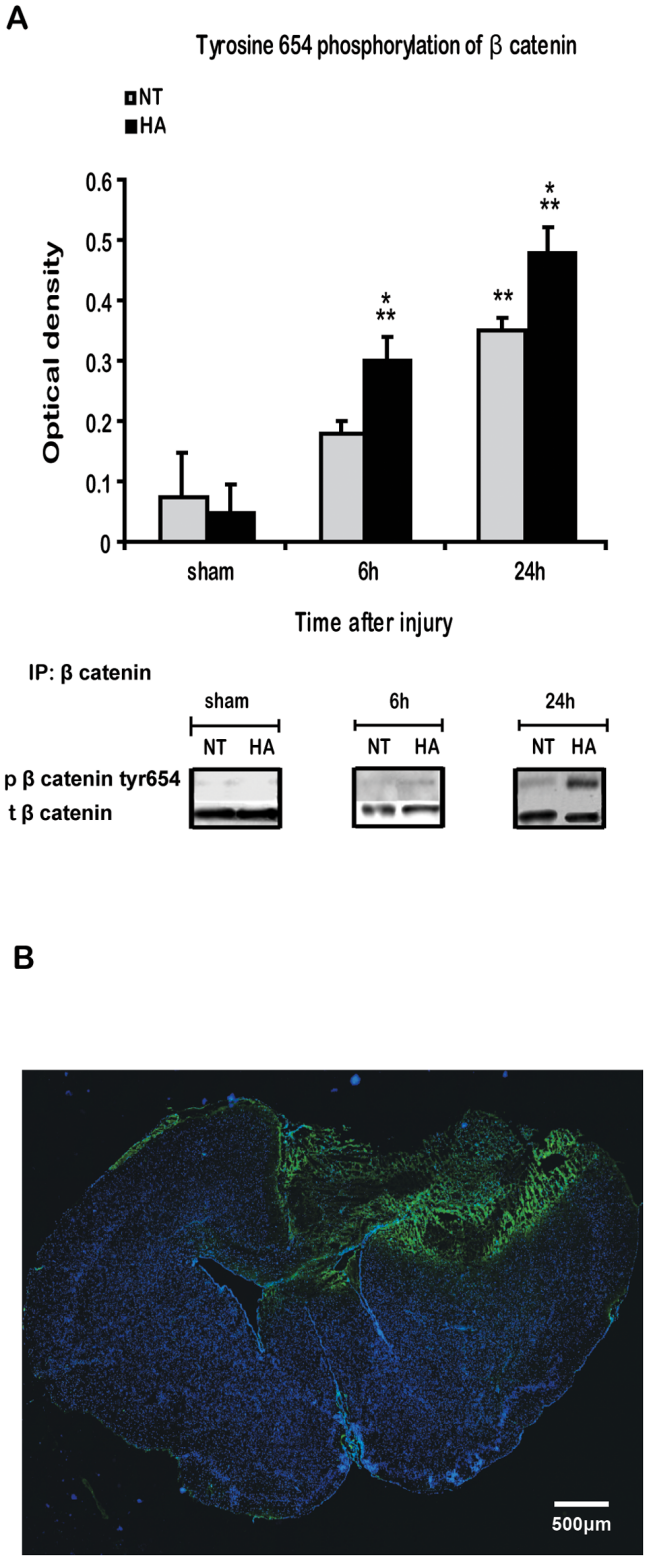
Heat acclimation induces tyrosine 654 phosphorylation of β catenin. Mice were subjected to CHI or sham operation, and injured cortexes were removed at 6 or 24β catenin, and Y654 β catenin was detected using western blots. Total β catenin was used as loading control (***A***). Immunostaining of the injured brain for Y654 β catenin (green) and DAPI (blue) shows induced Y654 β catenin phosphorylation restricted to injury area (***B***). Values represent the mean ± SEM. n = 5–6 per group. *p<0.05 vs. NT mice, **p<0.05 vs. sham mice, within the same group.

A band at 70–80 kD, under the predicted 95 kD band of β catenin was only observed after TBI (Figure 3D). This band was the cleaved fragment of β catenin described previously [24]. This fragment was already visible 6 h after injury in NT mice and was scarcely observed in HA mice. By 24 h the fragment was also detectable in HA mice, yet levels were significantly lower than in NT mice. Immunoprecipitation revealed that TBI also induces β catenin phosphorylation on the tyrosine 654 residue (Figure 4A). β catenin tyr- phosphorylation was significantly enhanced in HA mice 6 h and at 24 h after TBI. Interestingly, tyr-phosphorylation of β catenin was restricted to the injured brain area, as demonstrated by using immunohistochemical in Fig. 4B.

### Heat Acclimation Induces Increased Levels of N Cadherin and Detachment of N Cadherin- β Catenin Complex

**Figure 5:**
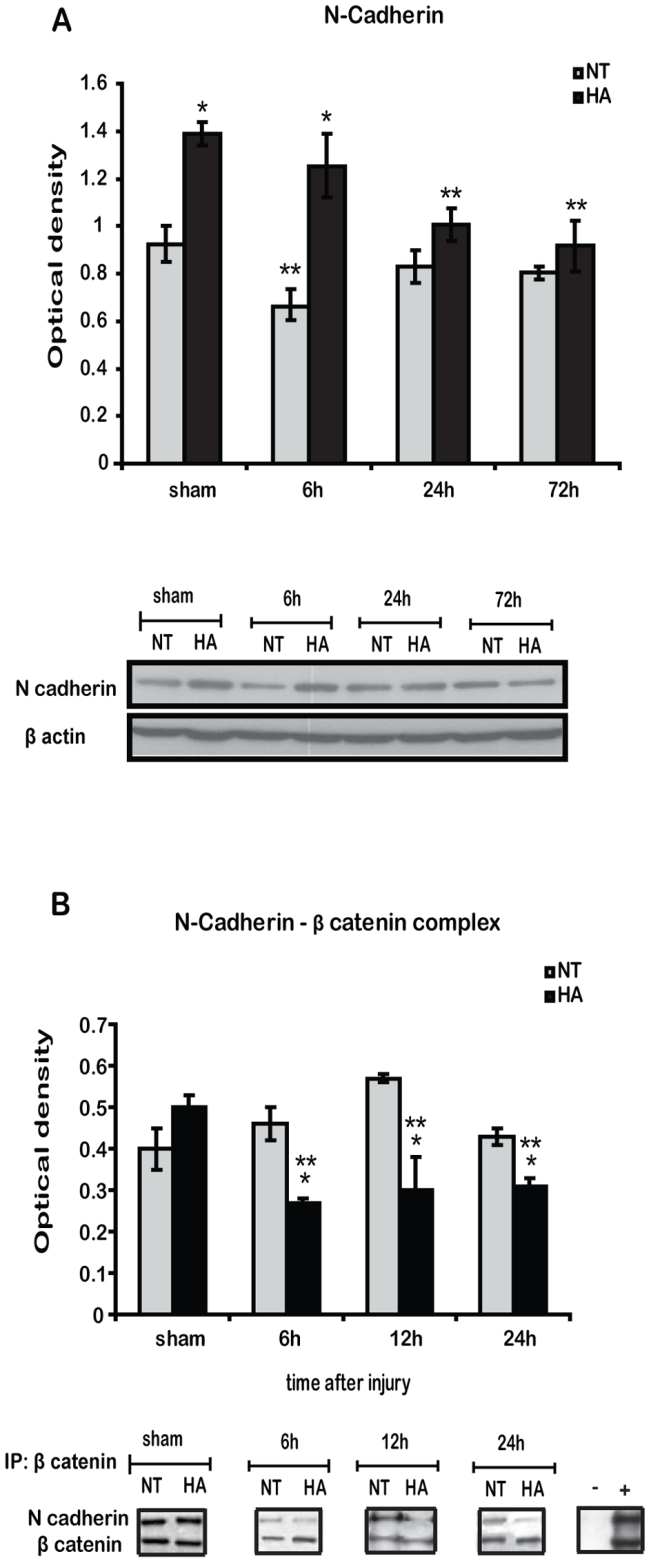
Heat acclimation induces basal N-cadherin and post injury detachment of the cadherin-catenin complex. Representative western blots of N cadherin, showing significant basal induction of N cadherin which lasts up to 6(HA) as compared with normothermic mice (NT) (*A*). β-catenin was immunoprecipitated (IP) with protein A beads and separated on SDS-PAGE gels. Immunoblotting (IB) of N cadherin indicates disassociation of cadherin-catenin complex in injured HA mice. (−) Immunoprecipitation of non β-catenin related protein (Bcl-xL) with the same beads. (+) input from non immunoprecipitation blot of 60 µg protein from injured HA brain (***B***). Values represent the mean ± SEM. n = 5–6 per group. *p<0.05 vs. NT mice, **p<0.05 vs. sham mice, within the same group.

Since tyrosine phosphorylation of β catenin regulates the integrity of the cadherin-catenin complex, we evaluated total N cadherin levels and cadherin-catenin complex integrity. N cadherin levels were up-regulated following HA as shown in Figure 5A, and higher levels were also observed 6 h post TBI, followed by a gradual decrease. TBI induced a transient reduction of N cadherin levels in NT mice 6 h after injury, and levels recovered by 24 h. Figure 5B demonstrates the N cadherin-β catenin complex measured by co-Immunoprecipitation. TBI induced detachment of the N cadherin-β catenin complex in HA mice, demonstrated by the decreased levels of N cadherin after β catenin Immunoprecipitation. In NT injured mice no significant changes were observed in complex integrity after the injury.

### Heat Acclimation Induces Late Recovery of Membrane β Catenin and Synaptophysin after TBI

**Figure 6:**
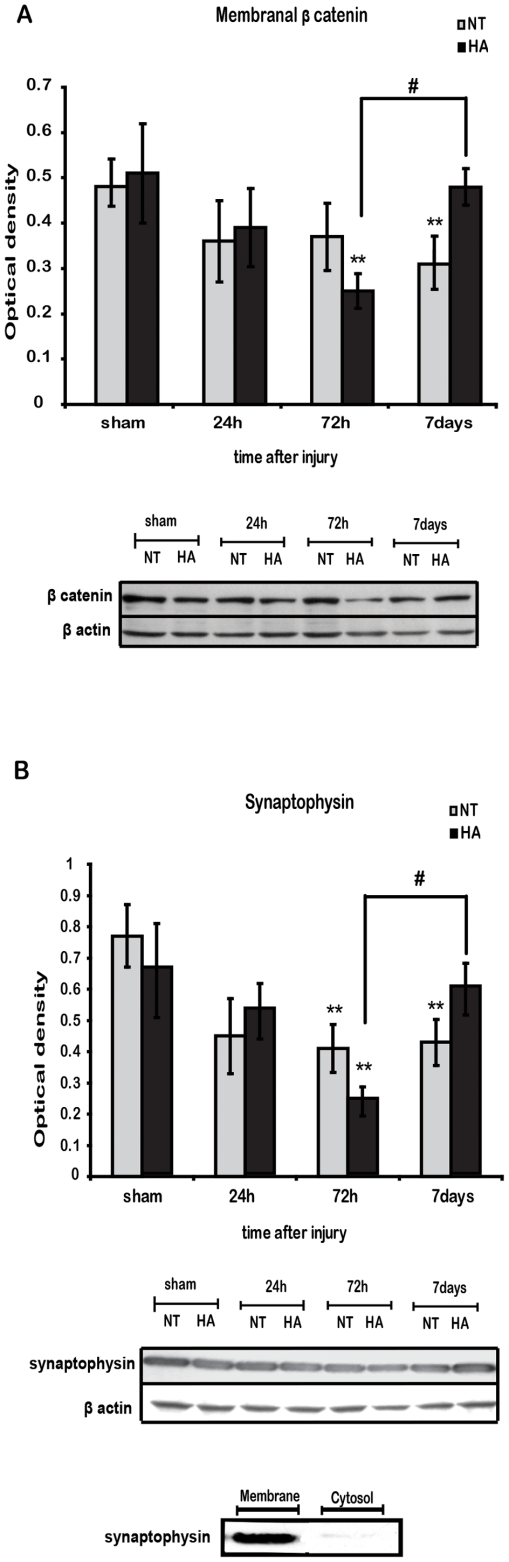
Heat acclimation induces transient reduction in membrane β-catenin and synaptophysin. Membrane cellular fraction extracts were separated on SDS-PAGE gels and analyzed using western blotting. After the injury a significant reduction in membrane bound β-catenin were seen at 72 h post injury in heat acclimated (HA) mice and after 7 days in normothermic (NT) mice. Membrane bound β-catenin levels were recovered by 7 days after injury (***A***) Blots of membrane extracts show significant reduction in synaptophysin levels in both groups at 72 h after injury with a recovery seen only in HA mice by 7 days post injury (***B***). The absence of synaptophysin in the cytosol verifies membrane protein enrichment. Values represent the mean ± SEM. n = 5–6 per group. *p<0.05 vs. NT mice, **p<0.05 vs. sham mice,within the same group.

Next we examined the levels of membrane bound β catenin, i.e. its role in cell-cell adhesion and synaptic establishment. Membrane bound β catenin levels slowly decreased in injured NT mice, reaching significance at 7 d post injury (Figure 6A). In contrast, in HA mice, its levels decreased more rapidly and transiently. The decrease was already significant 72 h after TBI and reverted to basal levels by 7 days. Protein levels of the synaptic marker synaptophysin were reduced 72 h following TBI in NT and in HA mice as shown in Figure 6B. However, sypnaptophysin levels recovered only in HA mice, at 7 days post injury.

## Discussion

The present study is focused on the role and the dynamics of β catenin after traumatic brain injury. β catenin is a member of the canonical Wnt signaling, regulated by GSK3β. Post traumatic alternations in this cascade can lead to apoptosis and depression in TBI victims. β catenin is also crucial for cell-cell adhesion as a part of the catenin-cadherein complex. Following TBI, interference in the complex can lead to motor and cognitive deficits. Here we show that following TBI, heat acclimation mediated cross-tolerance is reflected by attenuated motor disability and depressive like behavior in the injured mice. We provide evidence that in the acclimated - preconditioned mice, β catenin returns to form the catenin-cadherein complex, induces synaptophysin levels and, in turn, post injury synaptogenesis. These issues are discussed below.

### Heat Acclimation Provides Improved Motor Recovery and Reduced Depressive Like Behavior after TBI

Motor disability and depression are among the long lasting consequences of TBI. Our present findings corroborate our earlier reports on the beneficial effects of HA pre-conditioning on recovery of neurobehavioral function (measured using ΔNSS) [8], [9]. This study also demonstrates for the first time that HA minimizes depressive like behavior at 24 h and at 7 days after injury. The immobility time of NT mice in the forced swimming test increased after TBI, while the immobility time of HA mice was attenuated. In order to uncover the underlying mechanisms, we investigated molecular pathways involved in disability and depression in the injured brains.

### Heat Acclimation Induces GSK3β Phosphorylation after TBI

Akt is a “master regulator” of GSK3β and β catenin, independent of Wnt-mediated GSK3β inactivation. The PI3K/Akt pathway inactivates GSK3β via direct Akt-mediated phosphorylation of Ser 9, hence it inhibits β-catenin phosphorylation. This phosphorylation designates β-catenin for degradation and also prevents direct detrimental cellular activity of GSK3β such as apoptosis. Here we show induced GSK3β phosphorylation in HA mice, corresponding to the known enhanced Akt phosphorylation. Also, attenuated GSK3β activity partly explain the reduced apoptosis and attenuated pro-inflammatory cytokine levels seen in injured HA mice [7], [9], [30]. Consistent with other TBI studies [31], [32] GSK3β phosphorylation gradually increased over time only reaching statistical significance at 24 h in HA mice, suggesting that HA induces GSK3β inhibition after TBI. Akt phosphorylation peaks 4 h post injury in HA mice[7] implying that Akt may not be the main regulator of GSK3β inactivation. Other kinases linked to direct phosphorylation of GSK3β at ser9 are protein kinase A (PKA) [33] or PKC [34] that are likely candidates. Examination of the roles of these kinases was beyond the scope of the present study.

### Heat Acclimation Induces GSK3β-β Catenin Uncoupling after TBI

GSK3β is also partially regulated by the canonical Wnt signaling. The Wnt signaling includes GSK3β translocation from the active cellular complex that includes APC (adenomatous polyposis coli), Axin and CKIα (casein kinase Iα), or by phosphorylated LRP, keeping GSK3β away from its substrate. This allows β catenin stabilization [20]. Therefore, evaluating the phosphorylation state of GSK3β does not necessarily indicate kinase activity. Canonical Wnt signaling is traditionally measured by β catenin levels and the extent of its phosphorylation. Interestingly, although HA reduced GSK3β phosphorylation, the phosphorylation state of β catenin was unaffected by HA alone, with unchanged cytosolic levels of the protein after HA. However HA did affect β catenin dynamics in injured mice, with robust phosphorylation of β catenin at ser33/37/Thr41 observed shortly after injury and up to 24 h later, a possible result of down-regulated Wnt signaling in HA mice that does not involve GSK3β phosphorylation. Canonical Wnt signaling results in nuclear translocation of β catenin and cooperation in a transcriptional response mediated by its nuclear partner, T cell factor (TCF)-lymphoid enhancer factor (LEF) [20]. On the other hand, attenuated Wnt signaling allows β catenin to be marked for degradation which leads to a subsequent fall in cytosolic levels. However, despite massive phosphorylation at the positions known to mark β catenin for degradation, no significant changes were observed in the cytosolic levels of β catenin in HA mice. Our work is not the first to suggest that a model of neuroprotection inhibits β catenin^ser33/37/Thr41^ degradation. A similar phenomenon was documented under hypothermic conditions of focal brain ischemia [26]. All together, these findings seem to refute the concept that reduced Wnt signaling induced ser33/37/Thr41 β catenin phosphorylation obligates protein degradation, and there is possibly another pathway counteracting β catenin degradation.

### Heat Acclimation Inhibits JNK Phosphorylation after TBI

Recent studies suggest the presence of other kinases that phosphorylate β catenin at the same positions as GSK3β. Lee et al., [22] suggested that JNK, a member of the MAPK group, phosphorylates and regulates β catenin at the adherens junctions of keratinocytes at ser33/37/Thr41, thereby regulating cell-cell adhesion. Moreover, they also found uncoupling between ser9-GSK3β phosphorylation and β catenin cytosolic levels, in a manner similar to the current study. However, in our study the JNK hypothesis is ruled out by the fact that JNK phosphorylation was reduced in HA mice after TBI. Nevertheless, JNK inhibition after TBI may also contribute to HA mediated neuroprotection, as JNK activity after TBI is associated with neuronal death [35] and inhibition of JNK after TBI ameliorated neurological outcomes [36]. Furthermore, post injury hypothermia, previously found in HA mice [30] facilitates JNK inactivation and consequently reduces apoptosis [37]. JNK inactivation in HA mice may result from earlier Akt phosphorylation observed 4 h post injury [7],since Akt negatively regulates JNK activity [38]. Interestingly, JNK is a downstream factor in the non canonical Wnt signaling, that does not utilize β catenin as a signaling mediator, however the role of this pathway has mostly been investigated in cancers [39], and its implications in TBI have yet to be established. It is noteworthy that we evaluated kinase activity using phosphorylation levels rather than measuring direct activity, and there is not always a direct correlation between the two.

### Heat Acclimation Reduces TBI Induced β Catenin Cleavage

HA mice displayed robust phosphorylation of β catenin after TBI, however its cytosolic levels remained unchanged and there was no further degradation of β catenin. In order to reconcile this conflict we looked for evidence of β catenin cleavage. It is known that β catenin can be cleaved by cellular proteases including caspase 3, and the cleaved fragments of β catenin are seen at the molecular weight 70–80 kD in WB [24]. The cellular accumulation of the β catenin fragment following injury was significantly lower in the HA mice than in the NT controls. This may be related to our previous finding of reduced apoptosis and caspase 3 activity in HA mice after TBI [9]. However, the β catenin fragment may be derived from the activity of other proteases such as calpain [25]. Interestingly we did not detect any β catenin fragments in the nuclear extracts, indicating that these fragments do not play a role in transcriptional events. Neither cytosolic nor nuclear levels of β catenin were altered after the injury in both NT and HA groups, which may possibly imply that increased β catenin^ser33/37/Thr41^ degradation in HA mice is counteracted by the increased β catenin cleavage in NT mice. However this explanation is doubtful due to the relatively low amounts of the fragmented β catenin as measured by optical density, when compared with higher optical density of total or β catenin^ ser33/37/Thr41^. Increased β catenin^ ser33/37/Thr41^, as seen in HA mice, with no further alternation in cytosolic or nuclear protein levels may imply that HA blocks β catenin^ ser33/37/Thr41^ degradation. In that case, it is unlikely that the Wnt signaling plays an important part in the brain response after TBI or in the facilitation of HA induced neuroprotection. Another explanation may include the role of HIF1α. Studies suggest that under hypoxic conditions HIF1α can directly bind β catenin and regulate Wnt signaling [40], [41]. Based on our recent demonstration that activation of HIF1α plays a crucial role in neuroprotection of HA mice after TBI, it is tempting to speculate that such binding may protect the phosphorylated β catenin from degradation. However HIF1α-β catenin interactions were not examined in the present study.

### Heat Acclimation Induces Alternations in Post Injury Cell-cell Adhesion followed by Synaptogenesis- Does Tyrosine Phosphorylation of β Catenin Play a Role?

β catenin is crucial not only in the canonical Wnt signaling, but also in cadherin mediated cell-cell adhesion. The cadherin-catenin complex is localized in synaptic junctions and is involved in synapse formation, stabilization and plasticity [42].Here we report that HA induces up-regulation in N-cadherin, which remains after TBI, suggesting that these processes may be more effective after HA, as N- cadherin is important to cell survival, maintenance and to normal synapse activity [43], Moreover, N-cadherin stabilization is important in cell-cell adhesion and promotes activation of Akt and cell survival, as demonstrated in injured HA mice [7], [44]. N- cadherin levels are mainly regulated by endocytosis [43] and Ca^+2^dependent proteolysis [23]. Reduced levels of N cadherin after TBI may be attributed to higher intracellular Ca^+2^ levels after TBI and subsequent protease activation [45]. As a consequence of the reduction in the levels of N cadherin, together with alternations of β catenin after TBI, the cadherin-catenin complex maybe destabilized. Immunoprecipitation proved that TBI induced cadherin-catenin complex weakening and detachment of proteins in HA mice, in accordance to N cadherin levels. The alternations in β catenin that may interrupt cadherin-catenin complex are not restricted to β catenin cleavage, but may also be due to its phosphorylation state. There is evidence that tyrosine 654 phosphorylation of β catenin strongly regulates this complex [46]. Y654 phosphorylation of β catenin was recently linked to brain derived neurotrophic factor (BDNF), a well-known regulator of synaptic plasticity, morphology and neuronal survival [47]. Since BDNF levels are increased in HA mice after TBI [48], we were intrigued to evaluate Y654 phosphorylation of β catenin after TBI. We found that TBI induces an immediate increase in Y654 phosphorylation of β catenin in HA mice, with a delayed phosphorylation in NT mice at the same position. Interestingly, Y654 phosphorylation of β catenin was restricted to the injured area. The Y654 phosphorylation of β catenin is in agreement with cadherin-catenin complex integrity, which suggests that enhanced Y654 phosphorylation of β catenin precedes detachment of β catenin from the complex. Weakening the cadherin catenin complex is a natural process and is essential in the brain for synapse formation, synapse stability, axon outgrowth and guidance, these are accompanied by a reduction of β catenin levels at the cell surface but not in total cellular levels [49], [50].The phase of redistribution of β catenin and its re-association with N-cadherin is critical for memory consolidation processes [51]. We found that TBI reduces membrane bound β catenin levels up to 7 days after TBI in NT control mice, however with HA preconditioning, membrane levels of β catenin levels were transiently reduced and recovery was already noted 7 days post injury. This may imply new synaptic connections and improved memory consolidation ability. There is evidence that the cellular localization of β catenin has a role in synaptic regulation, and manipulation of Y654 phosphorylation of β catenin resulted in altered β catenin redistribution in the synapse and altered synaptic activity [51]. Therefore, enhanced Y654 phosphorylation of β catenin as seen in after TBI in HA mice may further support evidence for dynamic synaptic alternations in these mice. Interestingly, the levels of membrane bound β catenin correlated with the levels of the synaptic marker synaptophysin. Such a correlation may also suggest that enhanced synaptogenesis takes place in the HA mice and further supports the notion of β catenin involvement in this process. These findings together with our previous report on elevated BDNF in HA mice [48] a factor which is also crucial for synaptic plasticity and may require subsequent cadherin-catenin disassociation by tyrosine phosphorylation of β catenin [47] may suggest a mechanism to explain the enhanced hippocampal memory function found in HA mice after TBI [8].

## Conclusions and Remarks

**Figure 7:**
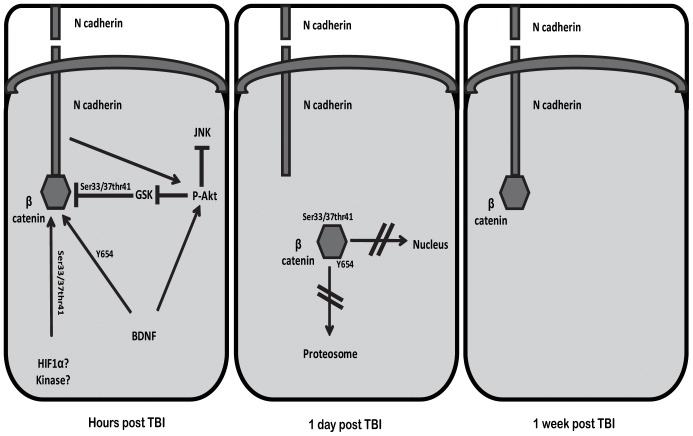
Schematic representation of suggested sequent of events determining β catenin fate in heat acclimated mice after closed head injury (CHI). In the first hours after CHI, basal high levels of brain derived neurotrophic factor (BDNF) and N-cadherin lead to abrupt increase of Akt phosphorylation upon CHI, resulting in inhibition of c-Jun N-terminal kinase (JNK) and glycogen kinase 3β (GSK3β). Inhibited GSK3β may lead to reduced ser33/37thr41 phosphorylation of β catenin later on. Increased ser33/37thr41 phosphorylation of β catenin implies on involvement of another player, potentially another kinase or hypoxia inducible factor 1α (HIF1α). Simultaneously, high levels of BDNF consequence in induced tyrosine 654 phosphorylation of β catenin, superior to the ser33/37thr41 phosphorylation which allows β catenin to escape degradation or nuclear translocation. Those events lead to weakening the catenin-cadherin complex in cell membrane, enabling synaptic changes at 1 day post injury. 1 week after the injury, β catenin translocates back to newly created synapses to form cadherin-catenin complex and establish cell-cell adhesion.

In conclusion, to our knowledge this is the first study to examine β catenin dynamics after TBI and to associate these dynamics with post injury synaptogenesis and cognitive function. This is also the first study to imply that dynamic changes in β catenin after TBI paly a role in synaptic alternations following TBI, in the same way, but on a different time scale to memory formation process [51]. We suggest that in the brain, under certain circumstances tyrosine 654 phosphorylation is “superior” to ser33/37/Thr41 phosphorylation of β catenin, enabling recruitment of β catenin for dynamic cell-cell adhesion and synaptic alternations following TBI. Figure 7 summarizes a theoretical sequence of events in HA mice after TBI: elevated levels of N-cadherin together with elevated BDNF levels lead to basally enhanced Akt phosphorylation. Following TBI, induced p-Akt inhibits JNK and may contribute to induced levels of inactive GSK3β, however it is unlikely to be the only kinase responsible for this inactivation. β catenin is phosphorylated at ser33/37/Thr41 with no further degradation or nuclear alternation. The kinase involved in this event is yet to be found. A non-kinase candidate is HIF1α that is capable of binding to β catenin thereby protecting it from degradation. Nevertheless, Y654 phosphorylation after TBI induces disassociation from N-cadherin, allowing synaptic changes after injury. At later time-points, β catenin is redistributed to the membrane and is possibly involved in synaptogenesis. Our study uncovered novel intriguing cellular events that may shed light on the neuroprotection conferred by HA.
